# Mining and Sustainability in the Circumpolar North: The Role of Government in Advancing Corporate Social Responsibility

**DOI:** 10.1007/s00267-022-01680-1

**Published:** 2022-07-14

**Authors:** Sarah Jackson, Gregory Poelzer, Greg Poelzer, Bram Noble

**Affiliations:** 1 University of Saskatchewan, Political Science, Saskatoon, SK Canada; 2 Luleå University of Technology, Political Science, Luleå, Sweden; 3 University of Saskatchewan, Geography, Saskatoon, SK Canada

**Keywords:** Governance, Arctic, Mining, CSR, Indigenous, Natural resources

## Abstract

Corporate Social Responsibility (CSR) is recognized as important to fostering sustainable natural resource development in the Circumpolar North. Governments are playing an increasingly active role in promoting and shaping CSR initiatives, often in collaboration with Indigenous communities and industry. This paper explores the role of CSR in mining for improving socio-economic and environmental management practice. The article argues that government instituted regulations can lead to the development and implementation of CSR practices by mining companies. To examine the relationship between government requirements and CSR, we use two Northern case studies: Cameco Corporation’s uranium mining operations located in Saskatchewan, Canada and Northern Iron’s iron mining operation located in Troms and Finnmark county, Norway. Through an in-depth review of scholarly literature, document analysis, and semi-structured interviews, our findings suggest that the role of the state in the initiation and implementation of CSR is of much greater importance than is currently acknowledged in the literature. In the case of Cameco, the Mine Surface Lease Agreements agreed to by the corporation and the provincial government provided motivation for the development and implementation of their world-renowned CSR practices, resulting in a community-based environmental monitoring program and benefits for both the company and surrounding communities. With Northern Iron’s operations in Kirkenes, working hour requirements instituted by the Norwegian Government allowed for significantly higher levels of local employment. Our findings suggest a greater role exists for government to facilitate the adoption of CSR policies, contributing in turn to improved socio-economic and environmental outcomes for Northern communities.

## Introduction

The Circumpolar North is experiencing significant environmental, socio-economic, political, and cultural changes, accelerating at an unprecedented pace (Bronen et al. 2020). Ensuring sustainable economic development while still addressing the wider socio-economic, cultural, and ecological implications of increasing resource exploitation remains a key challenge for Northern regions and communities (Fondhal and Wilson 2017; Young 2019). For most Indigenous communities, addressing this challenge requires building capacity for self-governance, retaining control over traditional lands and resources, and preserving traditional, spiritual, and cultural values (Gad and Strandsbjerg, 2019). Importantly, it requires that government and industry work with Indigenous communities to foster an environment of entrepreneurial growth and natural resource opportunities that are mutually beneficial, yet respectful of Indigenous peoples’ rights and interests (Long 2019; Nelson 2019). Responding to this longtime call for sustainable resource development in the North requires a stronger commitment to proactively pursue socially responsible actions that can attempt to correct decades of irresponsible resource exploitation in these regions (Gad and Strandsbjerg, 2019; Long 2019).

Corporate Social Responsibility (CSR) is one mechanism acknowledged globally, and across the Circumpolar North in particular, as important in the pursuit of sustainable and responsible natural resource development. Although variably defined (Loe et al., 2017), CSR is generally understood to include those activities that transcend a company’s core production activities and legally required behaviour, with focus on addressing the environmental and social concerns of stakeholders (Trebeck 2017). The basic concept of CSR is that corporations play a vital role in, and have both the power and the responsibility to, conduct their affairs in ways that satisfies not only shareholders, but accounts for the interests of stakeholders and promotes the well-being of potentially impacted communities. Within resource extractive sectors, CSR typically addresses such matters as environment management practice; social and community development; local employment and labour; and human rights (Campbell 2011). Proponents of CSR suggest adopting CSR standards have often resulted in positive improvements for local communities, satisfy economic growth, account for mitigation of potentially adverse environmental impacts, as well as promote improved health and well-being. Critics, on the other hand, have argued that CSR practices almost never live up to their aims and has only led to limited development benefits to local communities from resource extraction (Slack 2012; Frederiksen 2018). The mining industry is central to debates on the role of CSR in promoting sustainable environmental management and business practices (Higgins, 2014).

Growing use CSR in natural resource extractive sectors, particularly in mining within the Circumpolar North, is well documented in the literature (Kemp 2010; Slack 2012; Blowfield and Murray 2011; Franks 2015; Wanvik 2016, Frederiksen 2018). For decades, the mining industry devoted significant attention to CSR, seeking to address the outright negative environmental and social impacts of its operations. Yet, research on the sustainable development impacts of the mining industry’s CSR activities paints a bleak picture. Some scholars have argued that the voluntary nature of CSR has meant that companies are often unwilling to change their negative practices or invest in resources required to account for their impacts on local communities even after adopting CSR standards (Wanvik 2016, Frederiksen 2018). According to these authors, real improvement in industry practices will require stronger corporate accountability and the intervention of key stakeholders that can pressure corporations to behave in a socially and environmentally responsible manner (Idemudia and Kwakyewah 2018). Along these lines, there is now a growing debate on the need for a stronger regulatory role of government in encouraging industry practices in the natural resource sector that can foster responsible behaviour while forging respectful relationships with Indigenous communities (Steurer et al 2012; Larsen et al. 2018). The premise is that “governments represent a democratically legitimate and a potentially powerful stakeholder group that can define not only the scope of CSR by setting legal minimum standards but can also shape the meaning of CSR and promote respective management practices by using a variety of non-mandatory policy instruments” (Steurer et al. 2012, p. 207). Thus, the Circumpolar North serves as an excellent backdrop for investigating the effect of government decision making on CSR.

With the goal to examine the role of government in the initiation and implementation of CSR, which has to date received limited attention in the scholarly literature, our study poses two research questions:

1. How did government utilize policy instruments to steer corporate behaviour?
2. How were the policy instruments interpreted and implemented by corporations?

We explore how governments can utilize CSR initiatives to promote sustainability-oriented practices among mining corporations in the Circumpolar North. Activities ensured by government-enforced legal requirements cannot be understood to mean CSR; hence we emphasize the difference between outcome-based regulations and procedure-based regulations. Outcome-based regulations set targets, such as local hiring and environmental protection, that companies need to reach but are not prescriptive in how to achieve them (Hoberg and Malkinson, 2013; Natural Resources Canada, 2013). Such regulations are central to defining conditions set out in negotiated agreements between Indigenous groups and mining companies. Procedure-based regulations, such as Duty to Consult and Accommodate in the Canadian context and the Finmark Act in Norway, are understood to be CSR initiatives since they are typically not enforced by government but rather shaped by norms and the desire of companies to obtain a social license to operate (Jackson 2015; Wanvik 2016). For example, regulatory environmental impact assessments are not a form of CSR since they are instituted and enforced by the state. We analyze the impacts that procedural and outcome regulations have on the social, economic, and environmental management practices of two mining companies––Cameco Corporation (Canada) and Northern Iron Limited (Norway).

## Context

The need to advance effective CSR policies and practices within the mining sector has remained a topic of interest for decades. Scholars seeking to advance sustainability-oriented practices to guide the activities of the mining industry in the Circumpolar North have often pointed to CSR as an important concept that holds promise when it comes to addressing the complexities of mining development, including the severity and diversity of impacts caused by mining companies on northern environments and communities (Wanvik 2016; Yakovleva 2015; Ranangen and Lindman 2018). As a result of growing pressure to adopt more socially and culturally responsible practices that are guided by the principles of sustainable development, mining companies are now continually faced with the challenge to deliver on their corporate, social, and environmental responsibilities in the regions where they operate (Yakovleva 2015; Ranangen and Lindman 2018).

The concept of CSR was originally coined by two American scholars, A.A. Berle and C.G. Means, in the 1930s, shortly after the 1929 Wall Street Crash plunged the American economy into chaos (Klempner 2006). Modern popularization of the term, however, occurred in response to widespread criticism of the social and environmental practices of corporations beginning in the 1960s. Since the publication of the 1987 Brundtland Report, CSR has been heavily promoted by governments, non-governmental organizations (NGOs), inter-governmental organizations (IGOs), and corporations as a means of achieving sustainable development in an increasingly globalized world. Again, mining companies in particular were instrumental in disseminating the global norms of CSR and sustainable development through the policies of individual companies and through industry-wide initiatives such as the Global Mining Initiative (Dashwood 2014; Veiga et al., 2001; Yakovleva 2015). Although some have argued that CSR evolved out of a particular corporate-centered worldview that does not necessarily complement the diverse political and social arrangements within the Circumpolar states, its voluntary nature and the inherent flexibility of the concept have enabled it to be successfully applied in a variety of political and social contexts (De Geer et al. 2010; Argandona and Hoivik 2009a, 2009b; Long 2019). At the same time, CSR’s inherent flexibility makes it a difficult concept to define, and thus to measure. There are varied reasons for companies to adopt CSR policies and practices, and contextual differences that appear to influence the impacts and outcomes of CSR implementation, including differences in the expectations of companies (Amos 2018).

### The Role of Government in CSR

Traditionally, CSR debates are often centered around the effectiveness of CSR policies and practices, and criticisms of corporate action or inaction in ensuring sustainable resource development. The CSR-government relationship often seemed counter-intuitive to CSR proponents, based on the notion that CSR precludes a role for government and should remain in the realm of discretionary activities that operate external to regulatory environmental requirements (Gond et al., 2011; Dentchev et al. 2015; Idemudia and Kwakyewah 2018). Yet, there appears to be a renewed focus on the role of government, particularly within the context of the mining industry. Emerging debates suggest the need to deeply explore the CSR-government relationship to facilitate more responsible environmental and social development. The notion is that the role of “government” should be explored not just as regulators or facilitators of CSR; rather, “as key actors that can strategically use CSR for enhancing more sustainability-oriented development outcomes for communities” (Steurer et al. 2012; Dentchev et al. 2015; Wanvik 2016; Idemudia and Kwakyewah 2018). Further perspectives linked to these arguments assume that “government and not corporations are responsible for the well-being of citizens and can more effectively distribute the benefits of natural resource development in an equitable manner” (Jackson 2015). As repeatedly observed in the past, the distribution of the impacts and benefits from mining can often lead to the internal fragmentation of communities (Wanvik 2016; Idemudia and Kwakyewah 2018). Thus, as strategic facilitators of CSR implementation, governments are faced with the key challenge of developing policies and strategies that both encompass the voluntary nature of CSR but also are effective in meeting sustainability goals (Idemudia and Kwakyewah 2018).

A key perspective that needs to be addressed, however, is understanding the role governments should play in the implementation of CSR practices. Government involvement in the promotion and implementation of CSR can take several forms. A study by the United Nations Global Compact argues that policy choices available to governments in the context of CSR include:

- awareness-raising efforts to create a shared understanding of corporate responsibility among companies and the broader public, including what business can do to implement it;
- partnerships designed to create win-win situations in which various stakeholders work collectively toward a shared goal;
- soft law approaches that promote and incentivize voluntary action by business as a complement to state regulation; and
- mandating instruments that allow governments to monitor and enforce corporate accountability (Peters and Daniela 2010, p. 7).

According to Steurer (2010), governments are “expected to play four key roles in CSR: mandating (legislative), facilitating (guidelines on content, fiscal and funding mechanisms, creating framework conditions), partnering (engagement in multi-stakeholder processes, stimulating dialogue) and endorsing (publicity)”. Additionally, the Ministry of Foreign Affairs of the Netherlands explains that “governments play a key role in mediating between sometimes conflicting corporate and development agendas, explicitly spelling out priorities for developmental impact and providing guidance on how to reach CSR goals” (2013, p. 13). Thus, the implementation of CSR gives government the opportunity to promote their own development agenda in collaboration with corporations.

Governments equally receive several benefits from the effective implementation of CSR, for example engaging in CSR activities can assist governments in meeting broader environmental and societal needs (Institute of Medicine 2007, p. 1). It also enables governments to participate in “rethinking the role of companies in society” to achieve desired outcomes (Albareda et al. 2007). Notably, the discussion surrounding the role of government in CSR emphasizes the importance of flexible policies which enable CSR practices to be implemented effectively in different contexts, a perspective which complements the claim that CSR should remain voluntary (Albareda et al. 2007; Peters and Daniela 2010). Some scholars suggest that government involvement in the implementation and regulation of CSR may be growing, indicating the importance of further exploration of government involvement in CSR to promote the most informed and effective approaches to CSR practices ‘on the ground’ (Albareda et al. 2007; Dam 2012).

The role of government in ensuring effective implementation of CSR policies is particularly emphasized by scholars who argue that voluntary initiatives do not necessarily lead to effective practices, and that government regulation may be necessary to ensure just practices and the fair distribution of mining related benefits (Seck 2008; Knobblock 2013). Knobblock suggests that policies such as the “Minerals Resource Rent Tax” instituted by the Australian government in 2012 could provide a more balanced redistribution of the benefits obtained through mining (Knobblock 2013: 172). This suggestion is particularly relevant considering criticisms regarding the unequal distribution of impacts and benefits in developed contexts, such as northern British Columbia, Canada (Heisler and Sean 2013). Heisler nd Sean (2013) argue that the distribution of benefits by mining companies are used as political leverage in this context, to obtain a social license. Government policies such as revenue-sharing have the potential to ensure equitable distribution and avoid similar outcomes to those described by Heisler and Markey in the implementation of CSR. However, one caution in regard to revenue-sharing agreements is that the increased state involvement this entails may reduce the involvement of civil society in CSR, minimizing potential opportunities for community input in decision-making and the development of relations between companies and communities. To investigate the effect of government policy on CSR, we look at two cases in the Circumpolar North where mining operations offered the potential for new collaboration between companies and Indigenous communities.

### Institutional Context

Along with sharing many similar attributes of being Circumpolar North countries, such as climate, low population density, and natural resource development, to name a few, Canada and Norway share similar legislation and regulation in the governance of their mining sector. Both countries have minerals acts and environmental codes that evaluate the concession and its environmental impact, along with planning and building permits that fall under municipal responsibility (Poelzer, 2019; Fauchauld, 2014). In addition, their regulatory regimes account for the particularities of the context. For Canada, this meant recognizing Indigenous communities through the integration of consultation and input into decision-making processes, particularly resource development and land use. In Norway, legislation passed to protect the unique regional demographics, and subsequently interests, created space for regional decision making. We elaborate on these policy differences and the possible implications these have for government steering.

In the Canadian context, growing evidence of the adverse environmental, socio-economic, and cultural impacts of decades of resource extraction in the North has drawn considerable attention to the need for renewed oversight of the extractive industry (Stack 2012, Wanvik 2016, Frederiksen 2018). As part of reconciliation efforts to mend relations between the Crown and Indigenous communities, the Government of Canada has increasingly sought flexible governance mechanisms to help ensure the activities of industry promote sustainable development in these regions (Long 2019). Such renewed commitment to sustainability and protecting the rights and interests of Indigenous peoples as they pertain to resource development is further evidenced in the recent introduction of key legislation, by way of Bill C-15, that requires the Canadian government to take all measures necessary to align its laws with the declaration set out in the United Nations Declaration on the Rights of Indigenous Peoples (UNDRIP) (Jones 2020). A key implication of such legislation is that businesses and corporate entities pursing natural resource development projects in the North will be expected to conduct their activities in a manner that reflects UNDRIP commitments. As Long (2019, p. 3) explains, “industry and business play an extremely significant role in how the economic, social, and cultural aspects of reconciliation are addressed, including the extent to which opportunities and benefits are truly shared with Indigenous peoples and the environment of traditional homelands is safeguarded.”

Similarly in the Norwegian context, resource development, specifically the Alta Hydro Powerplant, led the government to assess its position on Indigenous rights. Norway ratified ILO 169 in 1990 which made it the government’s duty to help Indigenous peoples preserve their culture – including rights to their traditional land. This was followed up with Norway’s support for UNDRIP in 2007, which supports the socio-economic and political interests of the Sami population. At the domestic level, the recognition of Sami as Indigenous peoples is codified via Art. 108 of the Norwegian Constitution. In practice, this is through recognizing Sami rights and cultural-linguistic autonomy. Inherent elements of Saami traditional livelihood have included animal trapping, offshore fishing, shamanic practices and––above all––reindeer husbandry, a core part of Sami life and culture. Norway also established a Sami parliament in 1989 which bears responsibility for all matters pertaining to the Sami, in particular language, culture and politics (Szpak & Bunikowski, 2022). Most importantly, the combination of support for these UN initiatives and the recognition of Sami within the constitution translated to the adoption of the Finmark Act in 2005 which granted ownership of 95% of the Finmark Council under the management of the Finmark Estate Board. The Board is comprised of six members: half designated by the Finmark county council and half by the Sami Parliament. The Finmark Act, therefore, created a mechanism for northern residents in Norway to address the socio-economic challenges for the region.

The approach of both national governments to create avenues for community and regional development, that recognize the contextual needs, provide the backdrop for our investigation of the effect of government policy on CSR. Although both the Canada and Norway refer to international policy in Indigenous engagment, the differences in the focus on implementation and practice should result in varied government steering mechanisms.

## Methods

The study adopts Leslie Pal’s framework for public policy and policy analysis as the tool for examining the formulation and implementation of CSR policies in different contexts. Specifically, our analysis adopts the following theoretical perspectives put forward by Pal (2010):

- problem definition as a central element of a policy statement consisting of three components: reality (what is the unrealized needs or values), a desired state of affairs (what should be, the improvement), and the gap between them (the discrepancy);
- problem definition as being closely connected to the formulation of policy goals; and
- policy instruments as a means to address problems and achieve policy goals.

In line with these perspectives, CSR policies and practices are thus understood as policy instruments through which questions and concerns relevant to sustainable development and mining in northern communities can be effectively addressed. Our intent is therefore to examine the social and environmental challenges, including the opportunities facing Northern communities in relation to mining, a desired state of affairs which can be understood as achieving sustainable development in northern regions, and the gaps in terms of the discrepancies that exist between the CSR policies and practices of many mining companies.

### Case Studies

The article adopts a comparative case-study approach to examine the role of government in the initiation and implementation of the CSR policies of two mining companies: Cameco Corporation, which operates out of Saskatchewan’s Northern Administration District (NAD) in Canada, and Northern Iron Limited, which has one operating mine near Kirkenes in the far northeastern part of Norway (Table 1).

**Table 1:** Operational context for Cameco and Northern Iron Ltd

	Cameco corporation	Northern iron Ltd.
Location	Northern Saskatchewan	Northern Norway
Company Origin	Saskatchewan	Australia
Mineral	Uranium	Iron
Timeline	1988–present	2009–2015
Scale of Production	16% of world production	
Net profit margin	18.78	0.0300
Geopolitical importance	Surface lease requirements	Employment Protection Act
Local employment (%)	50% RSN employment 1500 Total employees	79% local employment 400 total employees
Local procurement	71% ($3 billion since 2004)	
Formal CSR Policy	Yes	No

Cameco Corporation is a well-established uranium mining company with operations in Canada, Australia, and Kazakhstan. The company was established following the merger of two crown (government) corporations - Saskatchewan Mining Development Corporation and Eldorado Nuclear Limited––in 1988, with its earliest operations based out of the Athabasca basin in northern Saskatchewan (Cameco Corporation 2015). Cameco’s presence brought significant economic development to the region, which in the past experienced challenges in terms of infrastructure and education. The company’s continued operation and the benefits accrued from its CSR practices are crucial to the future development of the region (Noble and Birk, 2011).

Northern Iron Ltd, on the other hand, is a small Australian mining company operating just south of the town of Kirkenes in the far northeastern part of Norway. The history of the town of Kirkenes is closely intertwined with that of Sydvaranger iron mine. From the early 20th century, the town developed around the mine and generations of residents were employed there until the mine’s closure in 1996. Following the shutdown of the mine, the region experienced a period of economic diversification due to tourism, government investment, and new business opportunities associated with its proximity to Russia and the strategic importance of the port of Kirkenes. Consequently, the re-opening of the mine in 2009 remained controversial, particularly in light of the financial challenges the mine faced during this period and until the company filed for bankruptcy in 2015.

While the operations of these companies differ in multiple ways, from the minerals they mine to the scale of their operations and the profitability of their enterprises, the regions in which these companies are located are similarly rich in natural resources. Northern Saskatchewan’s economy is heavily resource-dependent, focused on mining, forestry, and traditional economic activities such as hunting, fishing, and trapping (Northern Development Ministers Forum 2014). The mineral reserves in the region are substantial, with three uranium mines located in the Athabasca Basin accounting for all of Canada’s uranium production and approximately 17% of global uranium production (Northern Development Ministers Forum 2014). Finnmark’s economy is largely resource-based, centered on oil and gas, fishing, mining, and reindeer husbandry (Finnmark County Authority 2015). The county also has unrealized reserves of iron, copper, gold, silver, palladium, and platinum that offer substantial opportunities for expanded mining (Petterson 2010).

Both companies face difficulties common to the Circumpolar North, ranging from distance, isolation, extreme weather, and challenging social contexts, which make them ideal cases for comparison in the context of this research. Both regions are also experiencing demographic and socio-economic problems as a result of their unique histories and the uncertainty associated with resource development. The Northern Administrative District of Saskatchewan has a population of approximately 42,000, spread across 45 communities. The region has limited infrastructure, and its inhabitants have lower education levels compared to the rest of the province. Two thirds of the population are under the age of 35, and about 86% of the residents of the region are of Indigenous heritage (Government of Saskatchewan 2014; ICNGD 2015, p.8).

Finnmark county has about twice the population of northern Saskatchewan, with approximately 75,000 inhabitants across 19 municipalities (Finnmark County Authority 2015). Of all the Norwegian counties, Finnmark has the largest total land mass and smallest population. Similar to northern Saskatchewan, Finnmark County’s population is growing, primarily because of high birth rates and increasing immigration. Of the estimated 40,000 indigenous Sami that live in Norway, 25,000 are in Finnmark County (Minority Rights Organization 2005). According to Statistics Norway (2010), those Sami that are registered in the Sami census comprise 18.8% of the population of Finnmark County. In 2005, *The Finnmark Act* transferred 95% of the area of Finnmark County to the inhabitants of the region in response to Sami demands for rights to lands and water (Solbakk 2006). There is also a history of resistance to resource projects in Finnmark County, which complicates future mining development in the region.

### Data Collection

Data for this study were gathered from both primary fieldwork and secondary sources. Semi-structured interviews were conducted with key informants from government, mining corporations, as well as community stakeholders in Saskatchewan’s Northern Administrative District and Finnmark County. Interviews in northern Saskatchewan were limited by an influx of wildfires at the time of the field research. Despite these drawbacks, relevant information was gathered from industry and community representatives at a regional workshop. In 2015, a total of six interviews were conducted with key informants for the Saskatchewan case, representing Cameco Corporation, community leadership (e.g., LLRIB Consultation Office of the Lac La Ronge Indian Band) and the provincial government; and seven interviews for the Finnmark County case, representing Sor-Varanger Municipality, Kompetansenter community center, Sydvarangar Gruve AS, Northern Research Institute, and Barents Institute. The total number of interviews was small, reflecting the limited number of key institutions involved. The intent of key informant interviews was to identify institutional perspectives and to verify secondary-source data, as opposed to statistical representation or interview saturation per se. Interview data were thus cross referenced against available information in published reports and documents by government, industry, international organizations, and NGOs.

Interviews were recorded, transcribed verbatim, coded and thematically analyzed. Interview questions were framed in line with the primary research objective, to explore the role of government in the initiation and implementation of CSR. Questions explored the formal requirements for CSR and the socio-economic benefits of both organizations’ CSR practices, based on the following themes:

- formal requirements for CSR relevant to mining operations;
- relationship and collaborations between mining companies and communities;
- established corporate goals (e.g. employment, local benefits, environmental stewardship) and adherence to those goals;
- value of the mining company to the municipality;
- observed differences between the mining industry and the oil industry; and
- perspectives on the role of government in the implementation of the company’s CSR practices.

## Results

Results are presented in two main sections: the practice of CSR, followed by the role of government in CSR implementation. In each section, results for each case are first presented, followed by a comparative analysis and lessons learned.

### CSR in Practice

Overall, results show key differences in the CSR policies and practices of Cameco Corporation and Northern Iron Ltd. Cameco’s CSR policy and associated practices appear well-developed and further ahead, with the organization being internationally recognized for its effective CSR practices. Northern Iron, on the other hand, does not appear to have an official CSR policy yet the organization has adopted practices that demonstrate significant commitment to fulfilling its CSR objectives. Table 2 provides a comparative summary of the CSR policies and practices of the two mining companies.

**Table 2:** Legal requirements and CSR practices of cameco corporation and northern iron ltd

Theme(s)	Legal Requirements – Cameco Corporation	Legal Requirements – Northern iron	CSR Practices- Cameco Corporation	CSR Practices- Northern Iron
Corporate Policies/Codes of Conduct	▪ Not mandated	▪ Not mandated	▪ Official CSR Policy	▪ No formal CSR policy
Aboriginal Partnerships	▪ Employment – work towards 67% local employment ▪ Compensation - provide compensation for the loss of commercial income resulting from the lease of the land	▪ Working Environment Act- sections governing offshore rotation and working hours	▪ Cooperation with Aboriginal business development corporations to facilitate economic development ▪ Three impact benefit agreements ▪ Trappers compensation agreements ▪ 50% employment of northern residents ▪ Number one industrial employer of Aboriginal people in Canada	▪ Agreements with local Sami pertaining to potential mine expansion ▪ 79% local employment
Environmental Stewardship	▪ Environmental Impact Assessment (Federal and Provincial) and uranium mining legislation ▪ 1993 Joint Panel Recommendations	▪ Environmental Impact Assessment and the Pollution Act	▪ Community-Based Environmental Monitoring Programs	▪ No formal corporate policy or programs ▪ Only informal stakeholder engagement
Training, Education and Awards	▪ Employee education and training – maximize training and job advancement opportunities for northerners ▪ Education promotion – work with other companies, government and northern schools to plan and implement programs that encourage northern students to complete high school, pursue higher levels of education and consider professional careers related to the mine industry		▪ Academic awards & scholarships ▪ Counselling and wellness ▪ Employee and family assistance programs	▪ Norwegian language training
Labour relations	Employee services - provide employees with on-site services and counseling programs		▪ Fly-in, fly-out work schedule ▪ Network of northern air traffic pickup points for employees ▪ Apprenticeship programs ▪ Work placements	N/A
Community and Business Development	▪ Northern business participation – work towards a goal of northern businesses supplying 35% of total goods and services		▪ 71% local procurement of goods and services ▪ Community-focused impact assessments ▪ Regular meetings with local trappers and communities to assess the importance of traditional activities in relation to mining	▪ Local procurement of goods and services ▪ Opportunities for community consultation ▪ Risk assessments
Community participation	▪ Community vitality- assess community vitality challenges such as the social well-being and quality of life of residents ▪ Public involvement- consult with and inform northerners about their operations in northern Saskatchewan		▪ Cameco Northern tour ▪ Project-specific engagement programs and mine tours ▪ Over $14 million in donations to northern and Aboriginal groups since 2004 ▪ GIS mapping of traditional ecological knowledge	▪ Support for community activities e.g. local volleyball team

#### Cameco corporation

Cameco has a well-established CSR policy that has evolved over decades of its operations in Northern Saskatchewan. A primary objective for the organization is “to develop and maintain long-term relationships and provide communities with employment and business development opportunities and capacity building” (Cameco Corporation 2015). This objective embodies much of Cameco’s approach to CSR. The organization’s CSR team is responsible for the development and implementation of their five-pillar CSR strategy: workforce development, business development, community engagement, community investment, and environmental stewardship (Cameco Corporation 2015). Accordingly, the organization has over the years instituted several programs and agreements with local communities within it’s regions of operation. These programs range from academic awards and scholarships, apprenticeship programs and work placements, counselling and wellness, employee and family assistance programs, to supporting community environmental monitoring of the impacts of uranium mining operations.

English River, Southend, Lac La Ronge Indian Band, and Pinehouse are some communities in the Northern Administrative District which currently have formal collaboration agreements with Cameco. The organization has adopted a progressive approach to Aboriginal relations and has been recognized as the number one industrial employer of Aboriginal people in Canada. For example, about half of the employees at Cameco’s northern mine sites are residents of Northern Saskatchewan, and about 90% of these residents identify as Aboriginal. Cameco also employs northern businesses in supporting its operations in Saskatchewan, many of which are Métis or First Nations owned. These businesses, in turn, make it their policy to ensure they hire Aboriginal workers from northern communities (MAC, 2014, p. 2). Overall, available reporting suggests Cameco has been widely recognized for its effective CSR policy and practices (Mining Association of Canada 2020).

Important to note is that when it comes to CSR, there is often a gap between what companies say they do and what they actually do “on the ground”. Further insights into CSR implementation activities by Cameco revealed the organization has adopted several important strategies to enable the integration of Aboriginal workers in its operations. Among such strategies is “maintaining a seven-day in, seven-day out work rotation and a network of northern air traffic pickup points for employees. This system makes it convenient for northern employees to work in the mines one week and remain in their home communities during the next” (McIntyre and Cook 2002, p. 2). Cameco has also signed on to several community-based agreements. In 1999, it signed an Impact Management Agreement with the Dene communities of the Athabasca Basin which “provides the communities with workforce development and dedicated engagement programs, community investment funding, and mechanisms to collaborate around environmental stewardship” (Cameco Corporation 2015).

Aside from involving northerners in its core operations, the company also supports several environmental stewardship initiatives including the Athabasca Working Group, Northern Saskatchewan Environmental Quality Committee, and project-specific engagement programs. Cameco has also in the past extended its engagement practices to encourage community participation in environmental assessment processes and ongoing environmental monitoring activities. This includes an off-site community-based environmental monitoring program to monitor the impacts of uranium mining near local communities. Data are collected by community members during traditional land use activities (i.e. hunting, fishing) and samples analyzed by an independent government-based lab. The program was established in part in response to a government-led environmental assessment recommendation that the company more actively engage communities in uranium mining environmental management (see Noble and Birk, 2011). Regular meetings are also held with community stakeholders, including local trappers to share monitoring results and assess the importance of land use for traditional activities and impact of mining activities (Cameco Corp., 2015). Between 2012 and 2013, Cameco signed collaboration agreements with English River First Nation and the Metis community of Pinehouse to “establish a framework and guiding principles for long-term working relationships with the communities” (Cameco Corp., 2015). In addition, there are a number of Trappers Compensation Agreements which “encourage trappers to continue trapping, and provide them with a yearly cash distribution and, for some, an allotment of fuel” (Cameco Corp., 2015).

According to an interview participant from Lac La Ronge Indian Band’s Consultation Department, “Cameco is setting the bar in terms of engagement. No other company is even close” (Carriere, 2015). This participant outlined some effective initiatives undertaken by the corporation including hosting open houses, meetings with leadership and communities, and using first-language videos to communicate with members of Aboriginal communities. As stated by the participant, “this is a win-win for them because if they provide the necessary information, the community feels a level of comfort and security.” The importance of Cameco’s CSR activities in maintaining social relations was also echoed by a current employee of the company, who asserted that “local employment increases trust in communities since most people tend to trust their neighbors more than they trust government or industry.” For this participant, a sign of the effectiveness of the company’s CSR policies and practices is that “Cameco continues to be able to operate in a very controversial industry and that’s indicative of social license to operate” (Cuddington, 2015). As previously highlighted, CSR is also understood to be voluntary initiatives undertaken by companies to obtain SLOs. These perspectives suggest that Cameco’s approach to CSR has contributed to its ability to obtain and maintain an SLO.

#### Northern Iron Ltd

Results revealed that there are no formal CSR policies in place for Northern Iron Ltd. However, the Sydvarangar Gruve’s website detailed key strategic drivers guiding the organization’s operations. These include: safety, health and environment, productivity, reliability, competence and culture, and sustainability, but the extent of their implementation and effect are unclear. Noteworthy, is that Northern Iron also places significant emphasis on creating a “zero harm” culture in the workplace, stating that “the safety of our people is fundamental to our business…[and] requires the ongoing commitment of everyone in the organization” (Sydvarangar and Gruve 2015). While productivity and reliability do not closely relate to CSR but rather to branding and business practices, competence and culture can be linked to the organization’s social and economic CSR practices. A key focus for Northern Iron when it comes to CSR is “training employees into highly skilled and knowledgeable workers and developing leaders that can drive behavioural and cultural change”. The company further emphasize this perspective by stating that “investment in our people through training and development will be a driver to our future success” (Ibid.).

Throughout its mine operations, Northern Iron has offered several benefits to employees and community members alike. These benefits range from education for employees in Norwegian adult learning centers in Kirkenes to providing support for community social activities such as the local volleyball team and opportunities for community consultation advertised in the local paper (Barents Institute 2015). The company also entered into a collaborative agreement with local Sami pertaining to the potential mine expansion (Hermansen 2015). As one employee of the mine stated, Northern Iron’s “primary contribution to Kirkenes is tax money to employees and buying local products,” a statement indicating that despite the company’s lack of a formal CSR policy, it still contributes to economic development in the region through local employment and procurement.

The history of the Sydvaranger mine is, however, closely connected to public perceptions of Northern Iron’s CSR practices. The contrast between the benefits received from the town when the mine was state-run and the support currently received now that it is run by a small private company has in the past contributed to tensions between public officials, community members, and the mine. Shifts in tax law which have reduced the benefits to municipalities, as well as environmental concerns over the dumping of tailings in the local fjord have also contributed to tensions within the community in relation to the mine (Barents Institute 2015; Nilsen 2015; NORUT 2015).

#### Comparison of CSR Practices

Looking at CSR from a thematic perspective shows that both companies addressed nearly all the criteria in Table 2, except for Northern Iron which lacked programs for Environmental Stewardship and Labour Relations. While the scale of its activities was less significant than Cameco, Northern Iron demonstrated an understanding of the broad range of stakeholders in its endeavours. In particular, the agreements made between the respective Indigenous communities and the commitments to local procurement show an understanding from both companies around the importance of building partnerships intended to last the duration of the mine life cycle.

However, the scope and scale of Cameco’s CSR practices is significantly more substantial than Northern Iron’s, commensurate with the scale of each company’s operations. As a more established and larger entity, Cameco possesses more on-the-ground experience and resources to develop programs that fit the needs of its partner communities. Currently run as a small-scale private entity, Northern Iron has less capacity when it comes to the financial commitments and is limited in their CSR activities. Finally, a key difference comes with Cameco’s use of a formal CSR policy to guide its operations and decision-making while Northern Iron made references to drivers for its activities but did not create a CSR policy.

Further, despite Northern Iron putting attention into the same areas as Cameco, the ability for each company to reap the benefits of their work differed significantly. One factor central to these differences is the stage of the life cycle for Northern Iron’s Sydvaranger mine and the profit it produced. Facing lean conditions throughout the life of the mine, Northern Iron unsurprising put most of its attention to keeping the operation alive and as one interview participant at the Sydvaranger mine explained, “Northern Iron is no longer able to provide coffee to employees due to their low profit margin and subsequent financial constraints”. On environmental issues, Northern Iron relied on adherence to the laws and regulations as sufficient, which became apparent when the issue of dumping mine tailing into the fjord was raised with local environmental groups. When Northern Iron intended to double production, and thus waste, the increased dumping allowances required municipal approval which was initially rejected, then approved after some political deal-making. Because many residents were dissatisfied with the outcome, the legality of the decision needed approval from the County Governor. In dealing with this waste issue, Northern Iron only applied a minimalist approach, which may be necessary for a company struggling to stay solvent.

In the case of Cameco, where profitability enables more flexibility in decision-making, the effective implementation of a well-developed CSR policy can contribute to the reduction of tensions in the northern context. This observation was echoed by a representative of the company who stated that “Cameco has always had a high approval rating”. As of the fall of 2014, it was at 79%. Following the Fukushima disaster in Japan, the company still maintained a 70% approval rating (Dodson 2015). CSR makes good business sense for Cameco, reducing tensions, and providing the company with an exceptional reputation (Ibid.). A former employee of Cameco asserted that CSR has provided the company with significant business value including competitive advantage with regard to hiring and retaining northern employees, a strong track record within the industry, and the avoidance of costly regulatory delays (Dickson 2015). What seems clear in this context is that effective CSR policies, which clearly address social concerns have the potential to reduce tensions between the mine and community members.

### The Role of Government

Further examination into the role of government and its efforts in shaping CSR practices in both the cases of Cameco and Northern Iron revealed that legal requirements instituted by the state played a key role in facilitating the redistribution of social and economic benefits of mining operations to local northern communities. In the case of Northern Iron, the implementation of the Working Environment Act ensured a shift in the company’s hiring practices to include an increased proportion of local workers, seeking to comply with regulations regarding working hours. In Saskatchewan, the Mine Surface Lease Agreements (MSLAs) instituted by the Government of Saskatchewan ensured the redistribution of social and economic benefits to northern communities and equally played a major role in the development of Cameco’s world-renowned CSR policy and practices.

#### Cameco corporation – mine surface lease agreements

As identified, Cameco’s CSR policy evolved in significant part from the need to meet the province of Saskatchewan’s *Mine Surface Lease Agreements* requiring uranium companies to adhere to and be accountable for eight “*Northern commitments*” centered around maximizing training, employment and business benefits for local communities (Government of Saskatchewan 2016). The first MLSA was instituted by the Government of Saskatchewan in 1978. According to McArthur (2015), the agreements are designed to “address broader social and economic questions, such as worker health and safety protection, environmental protection, and the distribution of economic benefits”. In addition, mine operators are also expected to negotiate and enter into other *Human Resource Development Agreements* for each mine site. These agreements equally focus on “recruiting, hiring, training and job advancement opportunities for residents of Saskatchewan’s north and are signed collaboratively between the mine operator and the Ministry of the Economy” (Government of Saskatchewan, 2013: 2).

In line with the requirements of the MSLAs, mining companies operating in Northern Saskatchewan are required to make four northern commitments, with uranium mining companies obligated to make an additional four commitments. The northern commitments for all mining companies include: employment and job forecasting, employee education and training, business participation and opportunity forecasting, and compensation for income loss to a prior lease holder (Table 2). Additionally, uranium mining companies are required to commit to employee services, education promotion, community vitality, and public involvement (Dodson 2015; Government of Saskatchewan 2016). Companies are required to work towards 67% employment of northern residents and procuring 35% of their goods and services locally. Owing to low levels of education in the Northern Administrative District, fulfilling employment requirements has often proved challenging, particularly when attempting to account for the required 67% employment of northern residents (Dodson 2015). At present, approximately 50% of Cameco’s employees are northern residents and 71% of the goods and services utilized by the corporation are procured locally (ibid.). In all instances, mine operators are expected to report on fulfilling these commitments to the province to ensure accountability, measurement, and to enable the effective planning of future programs.

Cameco has been particularly successful in fulfilling these northern commitments having attained 50% employment of northern residents, while also instituting programs that aim to build capacity in order to increase the number of northerners employed beyond entry-level jobs (Dodson 2015). The organization is also committed to addressing community challenges around “social well-being and quality of life of residents” through its CSR initiatives such youth groups, a youth conference, and ongoing research on issues of interest to northern communities; for example, the impacts of fly in/fly out work rotations on families, challenges to post-secondary education and the like (Government of Saskatchewan, 2013; Cameco, 2015).

In 1993, the Federal Provincial Joint Panel on Uranium Development recommended the establishment of Environmental Quality Committees to create standardization and oversight of monitoring programs, in addition to building trust between northern communities, industry, and government. These committees gathered community input on mining operations and created a foundation for information sharing across all three parties. These committees precipitated the creation of the Athabasca Working Group (AWG) Environment Monitoring Program, which is today called the Community Based Environmental Monitoring Program (CBEMP). Every community in the Athabasca Basin participates in data collection on diet as well as community-based sampling programs for water, fish and wildlife. The data are collected by an independent Indigenous environment services company and samples are analyzed by the provincial government’s Saskatchewan Research Council.

What the results clearly indicate is that MSLAs have played a significant role in the evolution of Cameco’s CSR policy and continue to provide measures of accountability that facilitate effective planning and programming on the part of the corporation. Important to note is that while uranium companies in the region are expected to adhere to the northern commitments, the involvement of the provincial government does not take away the freedom and flexibility for the companies to achieve these commitments at their own pace and tailored to their desired approach. This creates an ideal context for the implementation of CSR: where companies are able to adapt to changing circumstances, while being accountable for their progress towards fulfilling their CSR commitments.

#### Northern iron Ltd – working environment act

Despite not having a formal CSR policy, Northern Iron is required to fulfill certain local employment conditions under Norwegian law. The Working Environment Act (2005), also referred to as the Employment Protection Act, outlines the regulations instituted by the Norwegian government relevant to working environment, working hours, and employment protection. Section 10.2 stipulates that “working hours shall be arranged in such a way that employees are not exposed to adverse physical or mental strain, and that they shall be able to observe safety considerations” (Directorate of Labour Inspection 2013: 26). Section 10.4 of the Act further stipulates that “normal working hours must not exceed nine hours per 24 and 40 h per seven days” (Ibid.). Although not intended for the purpose, these sections of the Act ensured that the town of Kirkenes received increased economic benefits from the presence of the Sydvarangar mine through high levels of local employment.

In practice, companies are required to maintain rotation of employees for limited periods during certain project activities such as the start-up of a mine. As highlighted, Northern Iron initially received an exemption during the start-up period of the Sydvarangar mine for the first two years of its operations. Following the exemption period, the company still had a significant number of its employees on offshore rotations - not only the long-distance commuters, but also the local workers. However, state intervention through the Labour Inspection Authority forced the firm to change its rotation system, ensuring full compliance with the working hour requirements outlined in the Working Environment Act (NORUT 2015). As summed up by researchers from the Northern Research Institute:

“The Employment Protection Act (Arbeidsmiljøloven) regulates working hours and shift/rotation. The law is relatively strict as a result of a strong tripartite cooperation between employers, unions and government. Shifts with particular long hours and long periods of “free time” are only permitted offshore (offshore rotation). Other industries can apply for exemptions from the Act for a maximum of two years. Start-up of a mine is an example, but after this exemption period passed, the company still had a major part of the workers with offshore rotation - not only the long-distance commuters, but also the local workers. Within a year they were forced by the Labor Inspection Authority to change the rotations system. It is now within the legal frames of the Employment Protection Act”(NORUT 2015).

Regulations governing working hours thus played a significant role in facilitating Northern Iron shift to more localized employment, seeking to adhere to the conditions of the Act. An employee of the mine pointed out that 79% of the mine’s employees are now local even though the proportion of local employees started off much lower (Hermansen 2015). Northern Iron’s shift towards increased local employment due to enforcement mechanisms once again demonstrates that government involvement can motivate corporate action, indicating the potential for government to steer CSR.

#### Comparative analysis

In both cases, the government utilized employment related policy as means to shape the effect of mining operations at the local level by setting targets for the mining companies. Importantly, the content of the MSLAs and the *Working Environment Act* differed based on the needs of the community. In Saskatchewan, the targets included broader socio-economic development while, in Norway, the targets placed more emphasis on working conditions. Each government recognized the needs specific to its jurisdiction and implemented corresponding policy targets that promoted socio-economic sustainability.

Cameco’s track record with CSR implementation highlights the important role of government as a driver of effective CSR policies and practices. Further, legal provisions adhered to by the corporation allowed for the development and integration of an internal corporate culture that included CSR as the core business approach, thereby resulting in net benefits for both the company and local communities. As one employee asserted, “Cameco’s CSR practices have resulted in value-added to the company through the development of positive community relations and a sustainable workforce” (Dodson 2015).

Legally enforced employment conditions led to greater overall benefits for Northern Iron and the communities despite the absence of a written CSR policy. While it may be out of scope to draw conclusions on the impacts of a lack of legal instruments in the Northern Iron case, what seems clear is that the role of state through legal instruments had a major effect in ensuring the redistribution of social and economic benefits in Kirkenes.

For environmental protections, the cases diverge significantly in their handling of community concerns and partnerships. For Cameco, the federal-provincial recommendations for establishing community-based committees to provide additional opportunities for input were adopted and developed not only to enhance the data for environmental decision-making, but to build trust between communities, the company, and government. Today, these committees are an established part of day-to-day business and a key component to the perception of Cameco’s operations. Northern Iron, on the other hand, only managed to meet the minimum environmental requirements regarding waste disposal and, further, needed special political approval to increase waste––without input or consent from local environmental organizations.

Both case studies demonstrate how legal requirements can ensure effective redistribution of the benefits arising from mining operations to local communities. One of the important characteristics of these CSR-style policies is the ability of companies to determine their path for reaching these requirements, giving them enough flexibility to incorporate them into their practices without compromising their fundamental business. For Cameco, this led to a shift in overall corporate practice that built a high level of trust with its partner communities. For Northern Iron, the lack of community engagement on important environmental decisions likely detracted from establishing a similar level of trust.

## Discussion

Although literature on the role of government in CSR has grown steadily, insights into the dynamics of government engagement with CSR remain insufficiently addressed. This article sought to address this gap by examining the role of the state, including the strategies employed, in facilitating the implementation of CSR policies and practices. Evidence presented in the case studies highlights the importance of government involvement in ensuring socially and environmentally responsible practices among industry. Instituting legal conditions helped not only in facilitating the adoption of responsible CSR practices by the companies, but also motivated Cameco Corporation to expand their practices beyond the legal requirements to engage communities in off-site environmental monitoring programs and provide greater social and economic benefits to communities than it normally would. This observation, however, does not necessarily negate the views of the many CSR proponents who stress the importance of CSR remaining voluntary and flexible in diverse contexts (Argandona and Hoivik 2009a 2009b; De Geer et al. 2010; Metaxas and Tsavdaridou, 2010; Cheshire et al., 2011). In instances where the role of government is emphasized, CSR scholars are primarily concerned with the implications of increased government involvement in CSR implementation (Steurer et al. 2012; Idemudia and Kwakyewah 2018). Some even argue that enforcement mechanisms are by definition ‘regulatory’ and do not necessarily fit under the CSR umbrella. This has prompted further debates on where the boundary really lies between regulation and CSR (Steurer et al. 2012; Jackson 2015; Wanvik 2016; Idemudia and Kwakyewah 2018).

For this reason, it is important to reiterate the importance of outcome-based regulations, which act as an enforceability mechanism, and their implications for understanding the ability for government to steer CSR. Legal requirements such as those demonstrated in the *Mine Surface Lease Agreements* and *Environmental Quality Committees* in Saskatchewan can provide measurable goals that companies can work towards achieving. Rather than restricting the ability of companies to respond flexibly in different contexts, these agreements allow the firms to work towards achieving their CSR goals in a manner that plays to the strengths of the company while simultaneously giving it greater insight into the challenging contexts in which it operates. As such, the actions of the companies undertaken in this vein are considered CSR rather than mere legal compliance. Thus, as illustrated with the Cameco case, approaches that allow governments to nudge companies toward creating strategic CSR‐related opportunities without putting restraints on them remain within the voluntary realm of CSR. Moreover, as Brammer et al. (2012) explain, CSR, even in its realm of self-regulation, is not completely independent of government intervention, be it in the form of collaborative (e.g. partnership with mining companies) or confrontational (e.g. naming and shaming) mechanisms (p. 4). The intent for government is often to both indirectly shape industry’s CSR practices and strategically ensure a level of accountability for companies to achieve predetermined outcomes (Idemudia and Kwakyewah 2018).

On the other hand, questions about the effectiveness of CSR continue to loom. While the cases demonstrate that a greater role for government may improve outcomes for northern communities, they equally shed light on landscape factors such as strong commodity markets and deposit quality as being crucial in determining the capacity of companies to provide effective CSR. In the case of Northern Iron, sharp drops in iron ore prices led to significant reductions in the company’s profit margin and, in turn, impeded its capacity to proffer benefits to the local communities in which they operated. Nonetheless, government involvement through legal requirements ensured the continued provision of benefits to local communities. In the absence of government intervention through CSR, the unequal distribution of the benefits of resource development could perhaps become the norm in the region. As communities across the Circumpolar North continue to experience the pressures from resource exploitation and climate change inaction, there is surely a need for stronger accountability and renewed government oversight over the activities of industry; CSR remains a key instrument at the government’s disposal. As Long (2019) points out, there is a need for businesses operating in the North to reconsider the nature of the social responsibility they bear to Indigenous peoples, and this can be accounted for within well-thought-out CSR frameworks.

Finally, there is a need for further research to examine the role of government and the institution of legal requirements to facilitate CSR implementation in other operating sectors and jurisdictions. A limitation of the present study is that it only examines two case studies with similar operational contexts. The study also focused largely on local employment including the redistribution of economic benefits as indicators of effective CSR implementation. Consideration of other sustainability indicators within and outside the northern context may shed a different light on the effectiveness of CSR and the implications of the role of state. This is particularly important given that there may be jurisdictions or regions where the strategies employed by government in the case of Cameco and Northern Iron may prove far less effective or may even be hampered by other institutional arrangements. Nonetheless, our findings provide a good starting point for understanding how effective CSR implementation through government involvement can result in corresponding social and economic benefits for northern communities.

## Conclusion

Although designed for companies to gain community acceptance, CSR is a powerful tool for reaching socio-economic and environmental outcomes. Therefore, CSR should not be relegated to a corporate practice for appeasement, but used to create conditions for long-term sustainability where government can set targets and encourage corporations to realize them. Finding a balance in the relationship between governments and corporations is necessary as they work together to address many of the impending sustainability questions around resource use and energy needs.

Since it focuses on environmental and social concerns (Trebeck 2017), CSR holds the potential to tackle complex issues that would be challenging for a government or company to deal with alone. The mining cases from Norway and Saskatchewan exemplify the potential for a government-led CSR approach that steers corporate practices to outcomes that work towards long-term sustainability. Further, because sustainability is tied to physical space, the issue of increasing overlap in land use is one area where finding sustainable outcomes is difficult, as is evident in many of the inconsistences within the UN’s Sustainable Development Goals (SDGs) (Gual et al. 2019; Xue et al. 2019).

Transforming our societies and economies to achieve sustainability objectives, such as the SDGs, is one of today’s greatest challenges; if unsuccessful the consequences for both people and the environment will be serious and far-reaching. Organizations such as the International Council of Science have expressed concerns about the incompatibility of socio-economic development and environmental sustainability (ICSU, 2015), a conundrum synonymous with mining. However, rethinking CSR as a tool for government to steer corporate practices shows promise in handling complex sustainability problems –– a development that is critical for future socio-economic and environmental vitality.
